# Rethinking deep learning in bioimaging through a data centric lens

**DOI:** 10.1038/s44303-025-00092-0

**Published:** 2025-06-26

**Authors:** Jiajun Cao, Jan Wenzel, Shanghang Zhang, Josephine Lampe, Hongxiao Wang, Jiachen Yao, Zhicheng Zhang, Shuo Zhao, Yu Zhou, Chao Chen, Markus Schwaninger, Jufeng Yang, Danny Z. Chen, Jianxu Chen

**Affiliations:** 1 National Key Laboratory for Multimedia Information Processing, School of Computer Science, Peking University, Beijing, China; 2 Institute for Experimental and Clinical Pharmacology and Toxicology, Center of Brain, Behavior and Metabolism (CBBM), University of Lübeck, Lübeck, Germany; 3 DZHK (German Research Centre for Cardiovascular Research), Hamburg-Lübeck-Kiel, Germany; 4 Academy for Multidisciplinary Studies, Capital Normal University, Beijing, China; 5 Stony Brook University, Stony Brook, NY USA; 6 VCIP & TMCC & DISSec, College of Computer Science, Nankai University, Tianjin, China; 7 Nankai International Advanced Research Institute, Shenzhen, China; 8 Leibniz-Institut für Analytische Wissenschaften – ISAS – e.V., Dortmund, Germany; 9 Faculty of Computer Science, Ruhr University Bochum, Bochum, Germany; 10 Department of Computer Science and Engineering, University of Notre Dame, Notre Dame, IN USA

**Keywords:** Computational biology and bioinformatics, Image processing

## Abstract

Deep learning has become essential in bioimaging for tasks. By examining data-centric strategies in general AI and revisiting existing deep learning methods in bioimaging, we describe a prototypical “BioData-Centric AI” framework. For AI users in bioimaging, this framework promotes a more practical approach beyond simply annotating large datasets or relying on a universal model. For method developers, it highlights key research directions to enhance AI toolboxes for the bioimaging community.

## Introduction

**Fig. 1:**
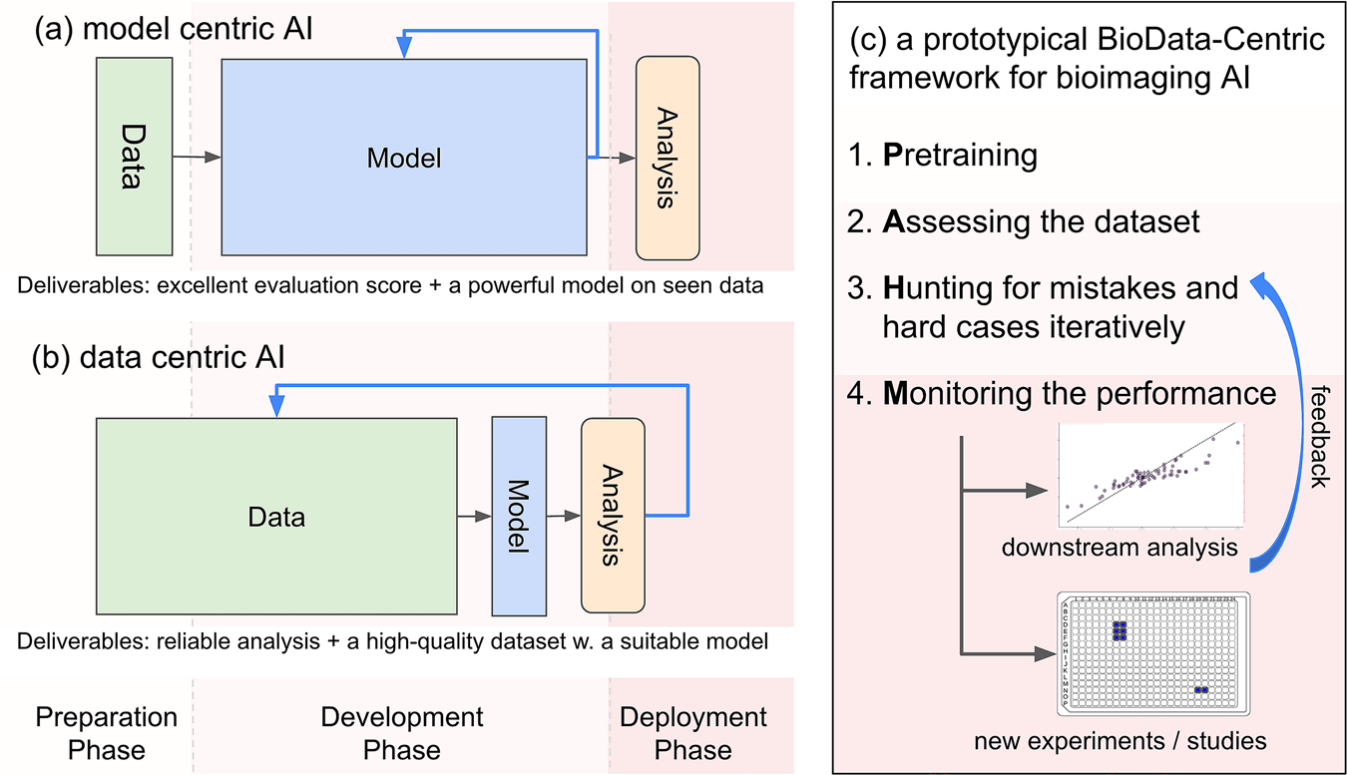
Model-centric AI vs. data-centric AI. Suppose we roughly divide the developmental process of a project into three phases: preparation, development, and deployment (marked by three different colors). **a**, **b** illustrate a model-centric approach and a data-centric approach, respectively. The relative sizes of the Model block and the Data block indicate the amount of effort invested in the corresponding parts. In the end, the model-centric approach delivers a powerful model with an excellent evaluation score on seen data. For the data-centric approach, the deliverables include a high-quality dataset (re-usable for future models) with a suitable model that can produce reliable analysis in practice, even on new data. A prototypical BioData-Centric framework for AI-based bioimage analysis is outlined in (**c**).

The rapid advancement of artificial intelligence (AI) has catalyzed a profound transformation within the field of bioimaging. Currently, there are two prevailing strategies in developing AI-based bioimage analysis methods: the model-centric and data-centric strategies (Fig. 1). In general, many endeavors align with the model-centric paradigm, where researchers iterate on models and algorithms against fixed benchmark datasets (Fig. 1a), such as the Cell Segmentation benchmark^1^, the Light My Cell challenge (https://lightmycells.grand-challenge.org/) and the CellMap challenge (https://cellmapchallenge.janelia.org/), to achieve higher evaluation scores over the state-of-the-art. These researches have undeniably played a pivotal role in motivating the development of cutting-edge AI methods, propelling progress from AlexNet to ResNet and the subsequent evolution into Vision Transformers, as well as nnUNet^2^. In contrast, the data-centric paradigm^3^ focuses on systematic data engineering, i.e., carefully preparing and refining datasets (e.g., data cleaning, de-biasing, etc.), usually with a fixed model architecture, to achieve the desired performance.

In practice, the data-centric strategy shows much more relevance in bioimaging (but unfortunately much under-explored) than the model-centric strategy, as many practical challenges in bioimaging do not directly originate from readily available benchmark datasets. In this paper, we want to rethink existing deep learning methods for various bioimaging problems with a data-centric mindset, as illustrated in Fig. 1b, from which we distill a prototypical BioData-Centric framework, as a conceptual guide on future tool and method developments. Different from the general data-centric AI, commonly referred as a discipline studying how to systematically engineer the data to build AI systems, BioData-Centric AI is a framework (1) that biologists can use as a template to conceptually design an AI solution for their real-world biological applications, and (2) where bioimaging AI method developers can use as a bridge to adapt state-of-the-art algorithm in general data-centric AI to the bioimaging field. In other words, the BioData-Centric framework is to use or adapt related data engineering techniques in general data-centric AI to solve biological problems, instead of studying and researching how to do systematic data engineering.

## A prototypical BioData-Centric framework for bioimaging AI

Distilling from solving various types of bioimage analysis problems in practice, a prototypical BioData-centric framework for AI-based bioimage analysis is depicted in Fig. 1c, including four key phases: **P**retraining, **A**ssessing the dataset, **H**unting for mistakes (development phase), and **M**onitoring the performance (deployment phase). The core concept is to leverage the large quantity of data effectively and to iteratively improve the model with minimal (but not zero) human intervention to obtain reliable analysis.

The initial step aims to give the model a robust starting point, either by utilizing an existing model for related problems or by allowing the model to learn directly from the raw dataset in a self-supervised manner, attaining a pre-trained model. The pre-trained model then serves as an effective “probe” to assess the dataset, e.g., enabling the gathering of an appropriate subset of data for initial fine-tuning of the pre-trained model in a supervised (or weakly supervised) manner. Here, such dataset assessment may involve identifying outliers (e.g., poor-quality images), detecting potential bias (e.g., severe imbalance in phenotypes), selecting the most representative images for expert annotation, etc. After the initial fine-tuning, it is important to efficiently identify potential errors, such as data samples with which the model struggles the most, so that human experts can curate such results, with minimal effort, and provide additional supervision to further fine-tune the model. This process can be repeated iteratively with a human-in-the-loop until the desired performance is achieved. Then, the model can be deployed, e.g., for different experiments or high-throughput studies, with performance monitoring for quality control, even in the absence of ground truth references. When potential issues are detected, further human-in-the-loop curation and fine-tuning could be performed to enhance the model continuously, embodying the concept of “life-long learning”^4^.

To illustrate this framework, we use a real microscopy image segmentation problem as an example that many computational biology groups may have encountered in a similar way. We seek to use this simple example (1) to explain how bioimage analysis problems could be solved with the BioData-Centric framework and (2) to point out important directions bioimaging AI method developers in our community could take for further investigations.

## An illustrating example: a vascular structure segmentation problem

### Problem description

Over 800 three-dimensional (3D) microscopy images were collected under different conditions to investigate the vascular effects and injuries that could occur in mouse models of diseases, after external influences, or upon genetic modifications. Each image is a *z*-stack of size 1024 × 1024 pixels along *XY* (one pixel = 0.51 µm × 0.51 µm) with 7 *Z*-steps of step size 4.25 µm (See Supplementary for details). Our image analysis goal is to accurately segment all vessels in all different conditions, which will then be used for 3D spatial quantification in morphology (e.g., branch density, thickness, branching point density, etc.) and topology (e.g., loops, voids, etc.).

**Fig. 2:**
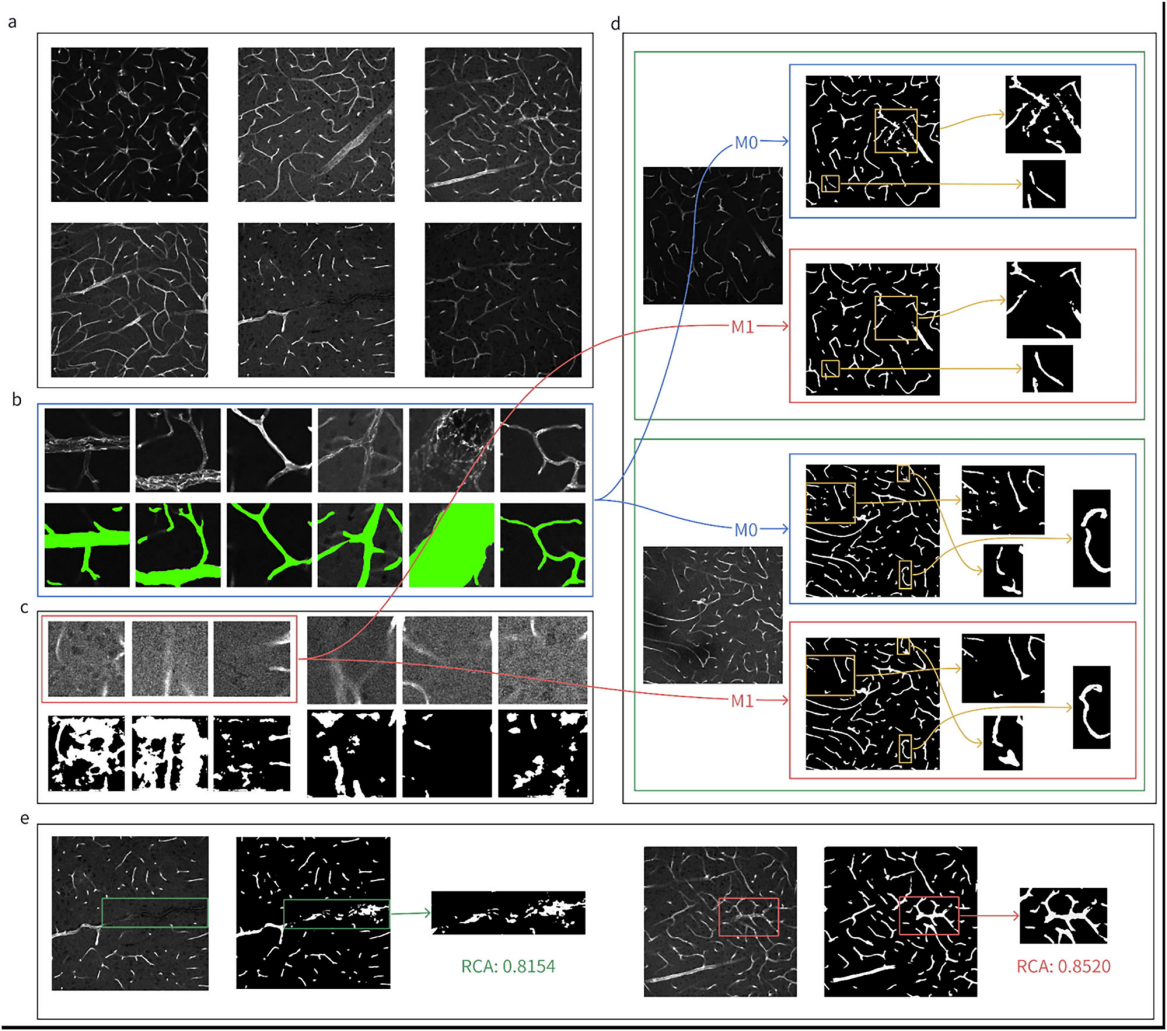
A vascular structure segmentation problem. All grayscale images or patches are displayed after Auto-Contrast in ImageJ. **a** Various image samples showing high diversity in the dataset. The size of each image is 7 × 1024 × 1024 (*ZYX*). **b** 25 representative patches (i.e., a core set) are selected from 90,860 patches to build model *M0*, 6 of which are shown here. Manual annotations are marked in green. **c** The top row contains image patches with high uncertainty. The bottom row shows the corresponding predicted segmentation. Three patches (within the red boxes) are manually selected from these image patches as the critical set to fine-tune model *M1*. **d** Segmentation results of model *M0* (blue boxes) and model *M1* (red boxes). Fine-tuning with the critical set significantly reduced model hallucinations. **e** Segmentation results evaluated using reverse classification accuracy (RCA). Two random examples are shown, one with relatively high RCA (right) and the other with relatively low RCA (left). Visual inspection can confirm that a higher RCA corresponds to a better segmentation result.

Considerable variations (e.g., different vessel morphology, different signal-to-noise ratio, etc., see Fig. 2a) in this large-scale data cohort make it impossible for a single classic vessel segmentation workflow (e.g., Frangi filter-based methods) to robustly work on all conditions. A deep learning-based solution holds great potential. Due to the limited number of z-slices in each *z*-stack, we chose to employ two-dimensional neural network models, which will be applied on the *z*-stack slice by slice. Considering the manual annotation cost, we divided our images into small patches of size 224 × 224 pixels for subsequent pre-training and training processes. At the beginning, there is no ground truth for training or no ready-to-use model (such as a pre-trained model or a foundation model). A current common practice in prevailing tools is manually annotating a large number of images and then training a deep learning model.

**Then, how can we approach this problem with the four-stage BioData-Centric framework?** It is important to note that the exact algorithms used here are all basic algorithms from the literature, selected intentionally to illustrate the core concepts. Further variations or more sophisticated solutions will be discussed in the next section.

- **Pre-training**: Roughly speaking, as long as the dataset to be analyzed contains more images than one can easily annotate manually, performing a pre-training on the entire raw dataset (or even including additional similar external datasets) is usually beneficial. Technically, in this example, we employed a Masked Autoencoder^5^ for self-supervised pre-training. This model learns to reconstruct masked parts of a raw image from the unmasked portions, allowing it to understand general patterns and features without manual annotations. The trained encoder will be used later in a specific segmentation model (TransUNet^6^ in this example) and further fine-tuned with a smaller annotated dataset to detect the structures of interest. This method reduces the amount of needed manual annotation by leveraging the knowledge gained during pre-training.
- **Assessing the dataset**: Not all data is equally important for a model to learn. If we allocate a specific time budget for annotation, say ten images, to train a model, there have been extensive studies showing that randomly choosing ten images to annotate usually yields much worse performance than carefully selecting the ten most representative images (so-called “core set”) to annotate. To select the core set in this example, we first applied the encoder of the pre-trained model on the entire dataset to map each raw microscopy image patch into a high-dimensional feature vector (e.g., dimension = 256) forming a so-called latent space. Two raw image patches are similar to each other means their corresponding feature vectors in the latent space are close to each other. By analyzing the latent space, we want to select *K* “hub” images, as the core set, which are (1) distant from one another but (2) close to the majority of the dataset in the latent space, so that the K images can represent the characteristics of the entire dataset. Technically, we employed the max K-cover algorithm as used in ref. ^7^ to select *K* = 25 image patches (from 90,860 patches in total) as the core set (see Fig. 2b), which were then manually annotated by human experts and used to fine-tune a TransUNet (with the pre-trained encoder) to get the initial segmentation model *M0*.
- **Hunting for mistakes and hard cases iteratively**: Even though the core set with 25 samples is very representative, it is still possible that the trained model may not be robust to all potential variations in the dataset. We could iteratively find more samples (image patches) that are “most challenging” (the so-called “critical set”) to the current model, and then curate them (i.e., inspect the segmentation results and manually fix segmentation errors if necessary) and add the curated data to the training set to improve the model. Technically, in this example, we employed a simple dropout algorithm as a Bayesian approximation^8^, which allows the model to generate not only segmentation but also an associated uncertainty value of the segmentation at each pixel. We then inspected 30 patches with the highest uncertainty and selected and curated three patches (as the “critical set”, see Fig. 2c) to enrich the training set. Then, we further fine-tuned the model *M0* into *M1* with the enriched training data. Figure 2d visualizes segmentation results from models *M0* and *M1*, demonstrating the necessity of error hunting and the effectiveness of fine-tuning on the “critical set”. It is worth mentioning that the visual comparison in this toy example emphasizes that visual inspection could be sufficient at a certain stage in certain applications. In reality, minor errors in the results of model *M1* could be further curated, i.e., may lead to *M2*, *M3*, etc., iteratively. It will depend on the downstream application whether we need a hold-out subset with manually annotated segmentation for evaluation (example quantitative evaluations on a benchmark can be found in the Supplementary). Here, we stopped at *M1* only for the demonstration purpose.
- **Monitoring the performance**: In practice, usually we do not have a large hold-out set with ground truth to validate the model performance, especially when new data are acquired, e.g., every month. From the data-centric point of view, we need to monitor the performance of the model when applied to images without ground truth continuously. Uncertainty, as in the previous stage, could be informative but is not necessarily correlated to accuracy in theory, due to potential mis-calibration^9^. Technically, in this example, we employed the reverse classification accuracy (RCA) technique^10^ to estimate the segmentation errors in an image without ground truth, as follows. For each new *Z*-stack (*z* = 7), say *I*_0_, we trained a simple Random Forest pixel classifier using the current segmentation of *I*_0_ (generated by the deployed model, i.e., *M1* in this example) and then applied the pixel classifier on the core set. Since we know the ground truth for samples in the core set, we can calculate the maximum accuracy of the pixel classifier on the core set, to represent the accuracy of the current segmentation of *I*_0_. The rationale for this approach is that if the current segmentation of *I*_0_ is reasonable, then a pixel classifier trained with such segmentation should yield reasonable results for those representative patches (with ground truth) in the core set. Sample results can be found in Fig. 2e, where a higher RCA value indicates better segmentation. If major issues are detected, additional annotations could be recruited to fine-tune the model.

## Beyond the simple example: further considerations and future directions

The aforementioned example aims to provide an intuitive illustration of our proposed four-stage BioData-Centric framework. Intentionally, we only chose simple algorithms in each stage to simplify the demonstration, which can definitely be further improved with more advanced techniques. The same framework can be realized for different problems with different variations, some of which have been explored preliminarily in the literature, while some require further investigation from method developers in our bioimaging community. In this section, we will revisit the four-stage data-centric framework from a broader perspective, comment on further technique considerations, and identify research gaps and future directions.

### Effective fine-tuning

Fine-tuning is a fundamental building block in the entire data-centric framework. Making the fine-tuning process effective also requires data-centric considerations. For example, new training data are collected by curating the critical set. If the new data are much smaller than the previous training data, simply fine-tuning the model with only the new data may cause losing the knowledge learned before (the so-called catastrophic forgetting, which could be potentially tackled with sophisticated algorithms^11^) and therefore cause overfitting to the new data. On the other hand, simply merging old and new training data may lead to ineffective training due to the imbalance in their sizes. Weighted combination, data augmentation of the new data, or representative sub-sampling on the old training set might be necessary.

The fine-tuning process could also be conducted using different techniques. The first example is dataset distillation^12^ or dataset quantization^13^, where a small “stereotypical” dataset can be generated when deploying the model to represent the knowledge where the model was trained on. Then, further fine-tuning can be easily performed on a combination of this distilled dataset and new training data. Second, AI-assisted labeling^14^ or weak labels could be a cost-efficient solution to reduce human efforts and therefore might help generate necessary supervision for fine-tuning efficiently. Weak labels refer to incomplete or imprecise labels, e.g., bounding boxes^15^, point annotations^16^ or scribbles^17^. Some pilot works on weakly supervised microscopy image segmentation have already shown promising results^18–20^.

### Pre-training

Transfer learning (TL) and self-supervised learning (SSL) are two primary approaches for pre-training. They share the same spirit: make good use of a large-scale (as large as possible) set of data, regardless of the availability of ground truth and the similarity to the microscopy images to be analyzed. The two approaches are suitable for different tasks and different types of data^21^. There is no conclusive study yet on a one-size-fits-all solution. As a rule of thumb, the decision mainly depends on the specific problem and resource availability. For example, when dealing with a nuclei segmentation problem, say, on a special type of cell not widely studied in the literature, TL from a pre-trained Cellpose^22^ model could provide a quick solution. On the other hand, when dealing with an in silico labeling problem on brightfield images and without public pre-trained in silico labeling models, TL from a pre-trained brightfield image segmentation model may not be ideal, as the two tasks may require different perceptions of the brightfield images. In this case, SSL was recently demonstrated to be very effective in improving in silico prediction performance^23,24^.

Different pre-training techniques have been developed for different types of models and different types of problems, e.g., pre-training for diffusion models for segmentation^25^, hierarchical pre-training^26^, and pre-training for video data^27^ (could be applied for time-lapse microscopy). As a community, having a collection of largely pre-trained models, like in the medical imaging field^28^, would be beneficial. Luckily, platforms like Bioimage Model Zoo are paving the path to this goal technically.

Finally, for certain problems, pre-training could also be done in a supervised fashion via pseudo-labels. Taking segmentation for example, this refers to “ground truth” generated automatically from another procedure (e.g., a classic image processing algorithm), despite it being error-prone. How to leverage the vast amount of pseudo-labels is still an active field in computer vision^29^, but not yet well explored in bioimaging.

### Assessing the dataset

Quality control on the dataset is important, especially for large-scale experiments. It might be necessary to clean the data before any analysis, such as removing duplicate records, mislabels, broken images, or poor-quality images, etc. There are several data cleaning tools in the computer vision community, like CleanLab (https://cleanlab.ai/), but not optimized for bioimages.

There are many different ways for constructing the core set^30^, among which one important consideration is the optimal core set size. Possible solutions include the classic ones, like Cohen’s d (effective size, see supplementary experiments) or more advanced refined core set selection^31^.

Quantitatively assessing the quality of a dataset has been a long-standing problem in machine learning^32^. One key metric is the diversity, as surveyed in ref. ^33^. A related metric on the opposite side of diversity is the bias in the dataset. Here, bias could refer to the amount of data from different sub-groups of the entire dataset (e.g., a rare phenotype in a large, diverse screening dataset). In this situation, data augmentation is particularly important in the BioData-Centric framework. This includes both basic operations, e.g., rotation or deformation, or more sophisticated strategies to mitigate bias^34,35^. Other de-biasing approaches beyond data augmentation also exist, such as adversarial training in clinical AI applications^36^, but not yet widely explored in bioimaging.

### Hunting for mistakes and hard cases iteratively

Besides using the simple pixel-wise uncertainty estimation method for constructing the critical set, there are different types of potential errors, defined by different types of uncertainty^9^. For example, for a thin curve- or tube-like structure, e.g., microtubule, errors in one or two pixels could be viewed as negligible by the model from a pixel-wise point of view, but could be critical in the topological sense when such “small” errors occur right along the curve (connect or disconnect). Thus, a topological uncertainty estimation^37^ would be beneficial. In addition, errors may also be “soft”. For example, in a mitosis classification problem, the model could make some very uncertain predictions on boundary cases, e.g., a cell near the end of mitosis can hardly be classified with a binary mitosis/interphase label. In this case, training and evaluation may need adjustment accordingly (e.g., training with soft labels^38^). In order to fix those mistakes, in practice, especially when dealing with extremely large datasets, seamless integration of user-friendly tools and effective uncertainty estimation would be critical to ensure the curation work is practically manageable. So, it would be great if common interactive platforms or tools, such as the human-in-the-loop component in Cellpose^39^ or the iterative curation in Allen Cell and Structure Segmenter^40^, could be equipped with sophisticated “hunting” algorithms.

### Monitoring the performance

Estimating the model performance on data without ground truth is still an open topic in the AI community. In the bioimaging field, it is still common to use the performance on a random hold-out set (with manual annotation when necessary) to represent the performance, but this could be problematic. First, this is only an average estimation over the given dataset (i.e., the dataset that the hold-out set is selected from), and cannot ensure catching individual failures or “outliers”. The RCA method used in the illustrating example provides a possible solution, but is still far from fully satisfactory in terms of computation efficiency and accuracy, and could be further improved (e.g., refs. ^41–44^). Second, this cannot represent the performance on newly acquired data (i.e., outside the dataset from which the hold-out set is selected). This is especially common in biological studies (e.g., extending the study cohort during paper revision). In this scenario, it will be important to analyze the domain gap between old and new data and detect out-of-distribution samples^45^ (e.g., initially studied on images of three types of cells, but later needing to include a new cell type with potential major morphological difference, or training an image restoration model on synthetic data but trying to apply it on real microscopy images, or training an in silico labeling model on images of fixed cells but trying to apply on images of live cells, etc.), where a special technique called “domain adaptation” or “test-time-training” could be used to improve the model performance. Domain adaptation is a widely studied topic in computer vision and medical imaging, and recently just started to be investigated in our bioimaging community^46^, while test-time-training improves the model’s generalizability at test time with self-supervision training and started to be tested in microscopic images^47^.

## Discussions and conclusion

### Model-centric AI vs. data-centric AI

As in the BioData-Centric framework described above, we believe the data-centric and model-centric approaches are not necessarily mutually exclusive and could actually be complementary in practice^48^. For instance, the training data quality and quantity accumulate over an iterative data-centric workflow. When the training set becomes considerable in size, certain model-centric concepts, such as AutoML or nnUNet, can be employed to further improve performance.

### Validation

The methods for evaluation and validation could evolve at different stages in the BioData-Centric framework. In practice, having a large hold-out set with ground truth is rare. In this situation, for example, visual inspection could suffice in the iterative “hunting for mistakes” stage. For quantitative metrics, common pitfalls have recently been summarized^49^. We could consider collecting special experimental data to quantify biological validity^50^. When applying the model to answer different biological questions, different metrics might be appropriate^51^.

### Requirements for efficient data versioning and management tools

Data-centric approaches generally involve many iterations of data addition, removal, or adjustments, therefore posing a great challenge in properly versioning the data and systematic management. In recent years, data version control tools have emerged, such as DVC (https://dvc.org/) and Git LFS (https://git-lfs.com/), making data versioning as easy as code versioning on GitHub. But, there is no product tailored for bioimage data, while some existing data storage infrastructures may not even be compatible with data versioning (e.g., due to limited storage space). We would encourage consideration of integrating data version control when planning the next generation of major bioimaging data management platforms and core facility data storage infrastructures.

### Foundation models

The rising trend of large foundation models has revolutionized, or sooner or later will radically change, how AI-based bioimage analysis works. We believe that if we can view foundation models from a data-centric perspective, the power could be further amplified. For example, the core of the new Segment Anything Model^52^ lies in over a billion annotated data obtained with a mix of various data-centric strategies (e.g., assisted manual labels and pseudo labels), which paves a viable path to build new foundation models, e.g., for general microscopy image restoration^53^ or universal in silico labeling, etc. Another issue is that when applying foundation models for fully automatic bioimage analysis, especially on a large scale of microscopy images, it will be important to automatically alarm potential failures, as discussed above. Beyond vision foundation models, a Multimodal Large Language Model that generates descriptive information to capture the biological features in images could also be an effective way to help researchers identify key attributes of the dataset or even automatically identify potential errors^54^. In a nutshell, the synergy between foundation models and data-centric AI will have the potential to redefine the bioimage analysis field.

In summary, we find that state-of-the-art data-centric AI algorithms in the broad machine learning community could shed light on improving bioimaging AI works in practice. When adapting general data-centric AI algorithms into the prototypical BioData-Centric framework, there are two key high-level adaptations and considerations:

- The many facets of “BioData” makes “systematic data engineering” a complex interdisciplinary task rather than an algorithmic engineering problem: for example, different biological validations for different problems at different stages (e.g., may need special web-lab experiments to collect validation data), consideration of the biological context (e.g., prior knowledge in biology or microscopy may affect implementation strategies), etc.
- The underlying biological application plays a decisive role in “BioData-Centric” AI: When should one start improving the machine learning model, instead of exclusively focusing on the data engineering? What types of mistakes are most critical to hunt for in the specific biological application? Which performance monitoring strategy is the most suitable one considering the specific biological assay? Etc. The key is to make sure it is “application-appropriate”.

Within this prototypical framework, we discussed major directions for consideration in practice and further exploration. We believe that revisiting any of the current bioimaging AI works through a data-centric lens will reveal new opportunities for further improvement from both the application and method development point of view.

## Supplementary information

Supplementary Information

## Data Availability

The implementation of the algorithms used in this example of data-centric workflow can be found at https://github.com/PKU-HMI/Data-Centric-Mindset-in-Bioimaging-AI.

## Glossary

AlexNet: One of the first artificial intelligence models that dramatically improved image recognition by using multiple hierarchical deep convolutional layers to extract visual features.
ResNet: A special artificial intelligence model that uses “shortcut connections” to train very deep neural networks effectively to achieve human-level performance on image recognition for the first time.
Vision Transformer: A special artificial intelligence model that analyzes images by breaking them into smaller patches and processing them in sequence to recognize patterns and features.
nnUNet: An artificial intelligence model that automatically adapts itself to segment biomedical images, enabling accurate identification of structures across various biomedical datasets.
TransUNet: An artificial intelligence model that combines convolutional neural networks with Transformers to accurately segment images by capturing both local details and global context.
Self-supervised learning: An artificial intelligence method where models teach themselves to recognize patterns within data by using the data’s inherent structure as guidance (e.g., by predicting one part of the data from another).
Foundation models: Large-scale artificial intelligence models trained on extensive datasets to serve as a base for various tasks.
Latent space: An internal representation where AI models encode data into a compressed form, capturing underlying patterns.
