# Supplementary material for: Rethinking deep learning in bioimaging through a data centric lens
Source: Npj Imaging. 2025 Jun 26;3:29. doi: 10.1038/s44303-025-00092-0 (PMC12202800; doi:10.1038/s44303-025-00092-0)
Supplement: Supplementary file 1 — Supplementary Information [file 44303_2025_92_MOESM1_ESM.docx]

## Rethinking Deep Learning in Bioimaging Through a Data Centric Lens

## Supplementary

**1. Code availability**

The implementation of the algorithms used in this example of data-centric workflow can be found at <https://github.com/PKU-HMI/Data-Centric-Mindset-in-Bioimaging-AI>.

**2. Experimental details**

A dysfunctional vasculature in the brain is considered to contribute to cognitive impairment (e.g., in aging or neurodegenerative pathologies). Specifically, we imaged cortical regions of mice that underwent an experiment to decipher the role of the protein NEMO in brain endothelial cells. Endothelial NEMO plays an important role in maintaining a functional blood-brain barrier. The dataset contains 814 confocal microscopy images (Leica, SP5, 20x immersion objective) from different mice either deleted for NEMO or not, and under different experimental conditions.

The images are of size 1024x1024 pixels along XY (one pixel = 0.51 µm x 0.51 µm) with 7 Z-steps of step size 4.25 µm. The choice of a z-stack with 7 z-slices is determined as an optimized balance, mainly between time and quality. Each tissue section was prepared with 100-µm thickness, while tissue processing, embedding and covering with a cover glass can lead to thinning of the tissue section. 7 z-slices with a step size of 4.25 µm permitted a good overview of most vessels in the tissue for quantification, even though not ideal for 3D visualization (highly anisotropic), and meanwhile not too time-consuming.

**3. Implementation details**

3.1 MAE Pretraining:

The Masked Autoencoder (MAE) pretraining involves reconstructing missing parts of the input data. The architecture consists of an encoder and a decoder. The encoder processes the visible (non-masked) patches of the input, while the decoder reconstructs the masked patches. The loss function is typically the Mean Squared Error (MSE) between the reconstructed and original patches：

$L_{\mathrm{MAE}}= \frac{1}{N}\sum_{i}^{N} \parallel x_{i}-y_{i}\parallel$ (1)

where $x_{i}$ is the original patch and $y_{i}$ is the reconstructed patch.

We used the MAE-ViT-Base as the pre-trained model, which contains 12 layers of Transformer encoders, an embedding dimension of 768, and 12 attention heads. The hyperparameters were set as follows:

- input_size: 224
- batch_size: 2
- mask_ratio: 0.75
- epochs: 400
- warmup_epochs: 40
- blr: 1.5e-4
- weight_decay: 0.05
- accum_iter: 4

There were 90,860 patches images with a size of 224x224 in the training data of vascular structure segmentation.

- 1. Core Set Size Selection:

The core set size K was chosen to balance computational efficiency and model performance. We chose to set K = 25, just as a demonstration example. As for how to determine the optimal size of the core set and how the size of the core set affects the performance, these are currently not within the scope of discussion in our article. Following [1], we have provided an effective method for core set selection, and the formula is as follows:


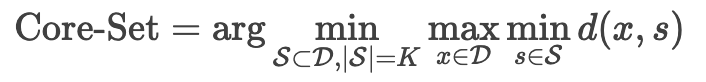


(2)

where $\mathbb{D}$ is the full set, $\mathbb{S}$ is the core set, and $d()$ is cosine distance

3.3 Details of Iterative "Hunting for Mistakes":

Monte Carlo (MC) Dropout was used to estimate model uncertainty by performing multiple stochastic forward passes with dropout = 0.1 enabled during inference. The uncertainty for a patch $x_{i}$ is computed as the variance of the model's predictions over T = 30 stochastic forward passes:

$Uncertainty(x_{i})=\frac{1}{T}\sum_{t}^{T} \parallel y_{i}^{t}-y_{i}\parallel$ (3)

where $y_{i}^{t}$ is the prediction for patch $x_{i}$ in the t-th forward pass, and $y_{i}$ is the mean prediction over T forward passes.

3.4 RCA Evaluation:

RCA (Reverse Classification Accuracy) is a method to evaluate the quality of model predictions by reversing the classification process. Instead of evaluating the model's predictions directly, RCA trains a secondary classifier on the model's predictions and evaluates how well this secondary classifier can recover the original ground truth labels. The idea is that if the model's predictions are of high quality, the secondary classifier should achieve high accuracy in reconstructing the ground truth. The RCA score of $x_{i}$ was computed as the accuracy of the Random Forest classifier $RF(x_{i})$, which was trained using $x_{i}$ and its prediction $y_{i}$ on validation set with ground truth labels:

$\mathrm{RCA}(x_{i})=\frac{1}{N}\sum_{i}^{N} (n_{i}=n_{i}^{RF(x_{i})})$ (4)

where N is total number of samples with ground truth labels, $n_{i}$ is the label and $n_{i}^{RF(x_{i})}$ is the prediction of $RF(x_{i})$.

The hyperparameters of the Random Forest classifier we used were as follows:

- n_estimators: 100
- max_features: 0.7
- random_state: 42

**4. Additional quantitative experiments**

To further demonstrate the task generalizability and applicability of the described BioData-Centric framework, we conduct an experiment on the cell nucleus segmentation task of the MoNuSeg dataset [2].

We split the training dataset into 333 patches of 224x224 and performed pre-training using MAE. Next, we selected the core set (K=27) and trained the model M0. Finally, we selected 42 patches as the critical set and trained the model M1.

Currently, the selection of the optimal size of the core set is still an active research direction, and manually setting the size as in the main experiments is a feasible example. In reality, a practical value would the maximum amount of annotation one can afford, or the as many as possible under a specific time budget. In this example, we adopt the Cohen’s d effect size to determine the scale of the core set. When the size of the core set is 27 patches, the Cohen's d effect size between the core set and the remaining set is approximately 0.8, indicating a large effect size.

To further illustrate the effectiveness of the framework, we additionally train a model (Fully Supervised) with the complete dataset and a model (Random) trained with data of the same scale as the core set but randomly selected as baselines for comparison with M0 and M1. Supplementary Table 1 shows the quantitative comparison, and we use MIoU and Dice as metrics. Supplementary Figure 1 shows the visualization of the qualitative comparison. Both in the quantitative and qualitative comparisons, we can observe that model M1, i.e., trained with the core set and further fine-tuned with the critical set, can achieve performance comparable to that of the fully supervised model with only about 20% amount of annotated data.

|  | Random | M0 | M1 | Fully supervised |
| --- | --- | --- | --- | --- |
| MIoU (%) | 77.0 | 78.0 | 78.6 | 78.9 |
| DICE (%) | 62.7 | 64.1 | 64.9 | 65.2 |

Supplementary Table 1. Performance quantification results of different baselines on the MoNuSeg dataset. The performance of the model (M0) trained with the core set surpasses that of the model (Random) trained with training data of the same scale but randomly selected. Moreover, the performance of the model (M1) further trained with the critical set is very close to the benchmark performance of the fully supervised model.


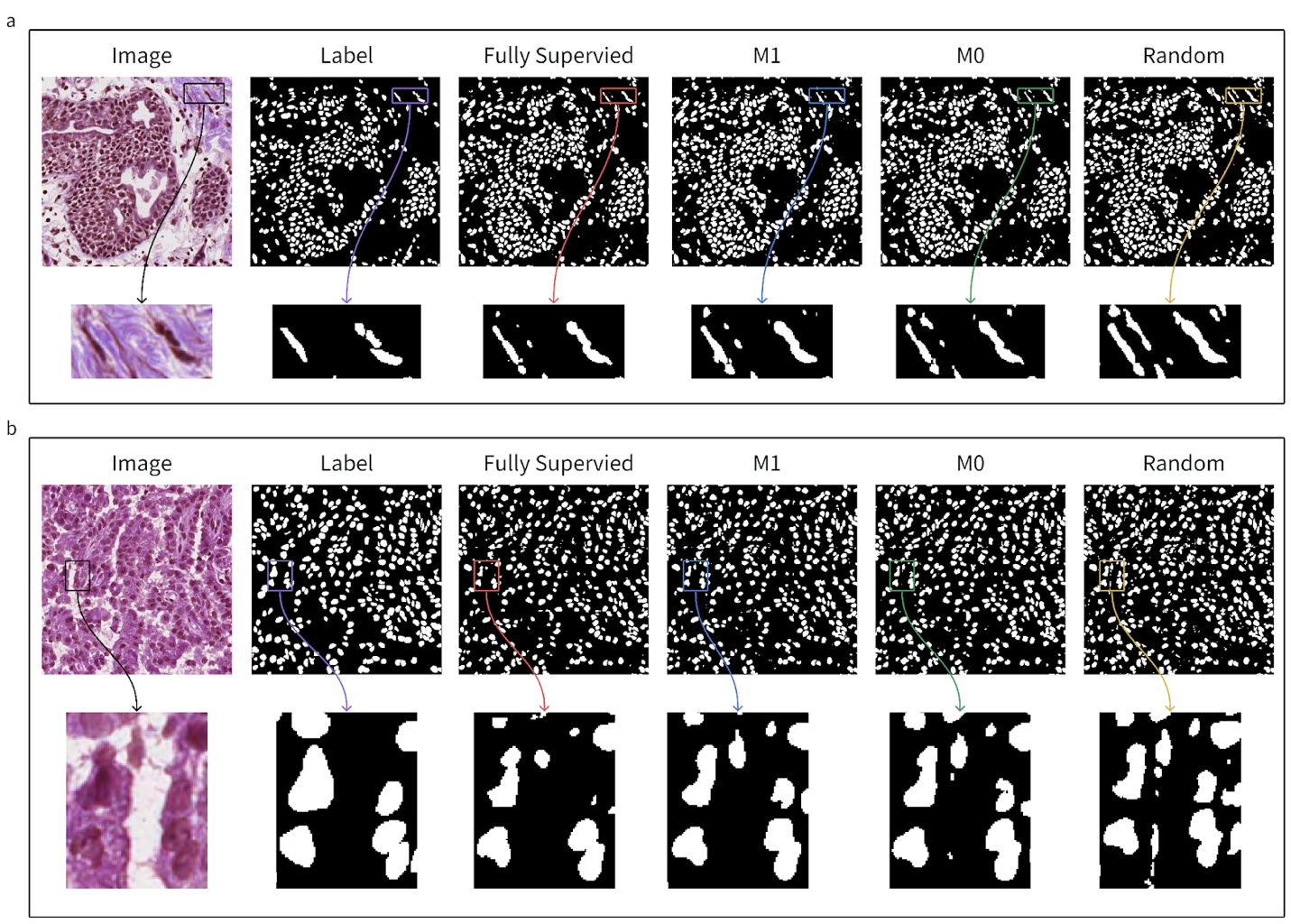


**Supplementary Figure 1.** Visualization of the segmentation results on the MoNuSeg dataset. (a) and (b) respectively show the segmentation results of cell nuclei in different tissue images. Among them, M0 and M1 represent the model trained with the core set and the model further fine-tuned with the critical set respectively. Random refers to the model trained with data of the same scale as that of the core set but randomly selected, and Fully Supervised refers to the model trained with all the annotated data.

References:

[1] Yang, L., Zhang, Y., Chen, J., Zhang, S. & Chen, D. Z. Suggestive Annotation: A Deep Active Learning Framework for Biomedical Image Segmentation. in *Medical Image Computing and Computer Assisted Intervention − MICCAI 2017* (eds. Descoteaux, M. et al.) vol. 10435 399–407 (Springer International Publishing, Cham, 2017).

[2] Kumar, N. et al. A Dataset and a Technique for Generalized Nuclear Segmentation for Computational Pathology. IEEE Trans. Med. Imaging 36, 1550–1560 (2017).
